# Finite element analysis of stress distribution in unilateral distal extension partial dentures: a comparison of four attachment designs

**DOI:** 10.1186/s12903-025-06213-w

**Published:** 2025-05-27

**Authors:** Maha Nagy Mohamed Kamal, Haitham Amr Mohammed, Abdallah Shokry

**Affiliations:** 1 Removable Prosthodontics Department, Faculty of Dentistry, British University in Egypt (BUE), Elshrouk city, Egypt; 2 Fixed Prosthodontics Department, Faculty of Dentistry, Fayoum University, Fayoum Government, Faiyum, Egypt; 3 Mechanical Engineering Department, Faculty of Engineering, Fayoum University, Fayoum Government, Faiyum, Egypt

**Keywords:** Unilateral partial denture, Extra-coronal attachment, Finite element analysis, OT cap attachment, Double OT cap attachment, Mini-bar attachment

## Abstract

**Background:**

Due to lack of consensus on optimal unilateral attachment retained partial denture design, this finite element comparative analysis study was conducted to measure the stresses induced from four different attachment-retained lower Kennedy class II removable partial denture (RPD) designs around the abutment teeth and the supporting residual ridge.

**Materials and methods:**

An educational cast of lower Kennedy class II having the first premolar as the last standing abutment was used. The abutment teeth - canine and first premolar- of the edentulous side were reduced to receive two splinted porcelain veneered metal crowns, while the intact side was prepared to receive a double Aker’s clasp and mesial rest seat for indirect retention. Then the cast was scanned, and the partial denture components and the splinted crowns were designed and assembled digitally using Blender4dental software. According to the attachment used; partial dentures were designed as follows: design 1: unilateral PD retained by OT cap attachment, design 2: unilateral RPD retained by double OT cap attachment, design 3: unilateral RPD retained by mini-bar attachment, and design 4: RPD retained by OT cap with major connector crossing the arch for stabilization (conventional RPD). The Meshmex software was used to modify the STL file, and the Abaqus software was used for finite element analysis. On the edentulous side, the first and second molars were subjected to vertical loads of 200 N and tangential loads of 23.5 N, while the first and second premolars were subjected to vertical loads of 140 N and tangential loads of 16.45 N. The von Misses stress levels induced around the abutments and at the residual ridge were measured and compared.

**Results:**

The lowest stresses were found with the conventional RPD design. Regarding von Mises stresses on the prepared abutments; The lowest von Mises stresses were found in design 4, followed by design 1, then design 3, while the highest von Mises stresses were found in design 2. Regarding von-Mises stresses on the residual ridge, the lowest Von Mises stresses were found in design 4, followed by design 3, then design 2, while the highest Von Mises stresses were found in design 1.

**Conclusions:**

The attachment retained RPD with a conventional design offered the lowest stresses applied for the abutment and the residual ridge. OT cap attachment exerts the least stress on the abutments when used in unilateral design RPD, so it is preferable for abutment preservation but contraindicated in weak ridges, however, the double OT cap exerts the highest amount of force on the abutment teeth and could be used with abutments showing perfect periodontal condition.

## Introduction

It is well known that Kennedy class I and II are always considered to be complicated dental situations especially in the lower arch, due to lack of support either from the palate or from the distal abutment; consequently, in order to reach appropriate and satisfying dental treatment with both functional and esthetic success, implant-supported PDs could be considered. Insufficient bone height and/or width and compromised medical conditions, besides the economic status of the patient, were the main factors that contraindicate the use of implants; however, the use of precision attachment to retain the RPD appears to be the accepted alternative treatment plan [1].

Dental attachment has two parts: a male part that is attached to the abutment crown or implant and a female part that is attached to the fitting surface of the prosthesis. Attachment-retained RPDs merge the advantages of fixed and removable prostheses considering adequate retention, accepted stability, improved esthetics, and proper distribution of masticatory forces to achieve functional and mechanical success [2].

Attachments could be classified in many ways; considering the method of fabrication, an attachment could either be semi-precision or precision. Attachment that allows some sort of movement between its parts is resilient; however, the rigid attachment prevents this type of movement. The location of the attachment in relation to the abutment gives us another type of classification: either the attachment is intra-coronal or extra-coronal [3].

Extra-coronal attachments with different designs are indicated mainly in free-end saddle cases with long edentulous spans that are considered a long lever-arm prosthesis combined with non-parallel short abutments where splinting is required for better stress distribution of masticatory forces and less torquing forces [4, 5].

Attachment-retained unilateral RPD design could effectively overcome the disadvantage of extensive major connector coverage needed in Kennedy class I, II. However, denture dislodgement during function due to the lack of cross-arch stabilization was noticed in many cases and could be avoided by proper selection of the attachment type and connecting of the abutments using splinted crowns [6, 7].

RPD retained by double OT attachment (OT unilateral attachment) system can be used in the form of unilateral design with no need for crossing the midline of the arch. The support obtained from this denture design and the way it is connected to the splinted crowns through the OT balls provides adequate stability during mastication, resembling fixed prosthesis-like function [8–10]. Double OT cap attachment was found to enhance the lateral stability of the denture with different degrees of retention provided by its retentive caps chosen according to abutments conditions, with overall functional and cost-effective solutions [11, 12]. On the other hand, it is documented that incorporating an extracoronal attachment to retain a unilateral RPD distributes more stresses over the distal extension ridge area with reduced stresses on the abutment teeth, which may be destructive to the edentulous ridge and lead to extensive bone resorption [13].

Bar attachment has the advantages of decreasing rotation of the denture base during use, enhancing denture support with better mastication, and reducing the load transmitted to the residual ridge under the denture base. Bar design attachment provides adequate retention and stability with increased prosthesis rigidity. It is considered a conservative surgical and economic line of treatment for partially edentulous cases [14, 15].

Many studies emphasize that, bar attachment could successfully be used for implant splinting or with bounded partial denture designs [16, 17], however no studies indicated the use of bar attachment in free end saddle cases as *Kennedy* class I, II.

Finite element analysis (FEA) is considered a valuable invitro method that offers unique advantages as a controlled, mechanistic tool to investigate biomechanical behavior as stress distributions under specific conditions that are difficult to isolate in clinical trials due to ethical constraints, patient heterogeneity, or practical limitations [18–19].

This study was conducted in order to measure the amount of stresses induced by different unilateral designs of extra-coronal attachments (OT cap, double OT cap, and mini bar attachment) retained Kennedy class II RPD on the abutment teeth and the supporting residual ridge compared to the conventional bilateral cross-arch design attachment retained RPD to answer the question: Is the attachment retained unilateral RPD design capable of replacing the bilateral design with the same or less stresses on the supporting tissues?

**Aim of the study**.

To compare the effect of using four different designs of lower-class II partial dentures when applying occlusal-like forces on the stresses induced around the abutment and the supporting residual ridge using finite element analysis.

## Materials and methods

The study was approved by the Ethical Committee of the British University in Egypt (BUE) with research approval number 24–046.

Method of model creation: an educational epoxy resin cast of lower Kennedy class II having the first premolar as the last standing abutment was used. First, the canine and the first premolar of the edentulous side were reduced to receive two splinted porcelain veneered metal crowns, while, at the intact side, double rest seats on the first and second molars to accommodate for a double Aker’s clasp and mesial rest seat on the first premolar of the same side for indirect retention were prepared.

The prepared cast was scanned using a desktop scanner *(3Shape E2 with 1920 × 1080| 1920 × 1200 resolution).*

The removable partial dentures were designed according to the extra-coronal attachment used as follows:

Design 1: unilateral RPD retained OT cap.

Design 2: unilateral RPD retained by double OT cap.

Design 3: unilateral RPD retained by minibar.

Design 4: RPD retained by OT cap with a major connector crossing the arch for stabilization (conventional group).

**Fig. 1:**
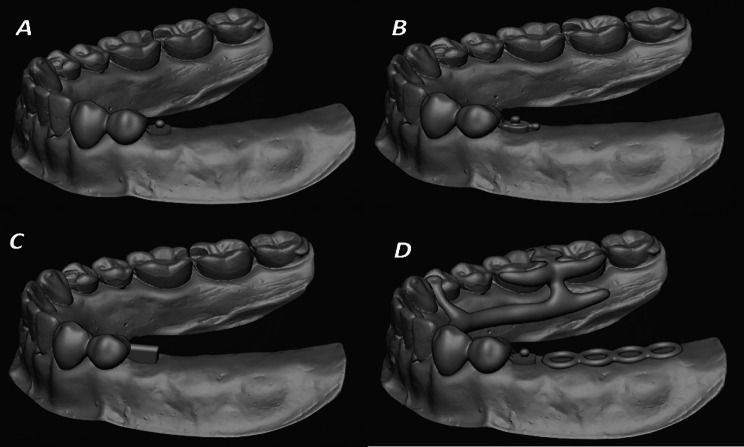
**A**; 3D model of the splinted crowns attached to the OT cap. **B**; 3D model of the splinted crowns attached to the double OT cap. **C**; 3D model of the splinted crowns attached to the mini bar. **D**; the bilateral design of the RPD

Porcelain-veneered, splinted metal crowns of the reduced abutments of the edentulous side were digitally designed and surveyed with a ledge prepared on the lingual wall and a rest seat prepared on the mesial side of the occlusal surface of the first premolar. Then the male part of the extracoronal attachment (according to the previously mentioned designs) was attached to the distal surface of the premolar abutment tooth crown digitally to ensure parallelism. Figure 1 (A, B, C, and D). Then the components of the removable partial denture framework design were added digitally, followed by the setting of teeth on the edentulous ridge, and then the female part of the extracoronal attachment was attached to the fitting surface of the denture base digitally. Finally, the four treatment option denture designs were obtained and saved to the software (Blender4dental).

Finite element analysis (FEA) was conducted on the four unique denture designs to evaluate their structural and mechanical properties under simulated conditions. The process began with obtaining STL files for each design, which served as the foundational geometric data for the analysis. These files were imported into Meshmixer software, a tool used for processing and refining 3D mesh models. In Meshmixer, the raw STL files were converted into solid parts, ensuring the models were watertight and free of defects. Additionally, volume was added to the designs where necessary to enhance their structural representation and ensure they met the requirements for finite element modeling. After completing the preprocessing steps, the solid models were exported to Abaqus software, a powerful tool for performing advanced finite element simulations. Within Abaqus, the designs underwent a meticulous discretization process, where the continuous geometry was divided into smaller finite elements to facilitate numerical analysis. Quadratic tetrahedral elements of type C3D10 were chosen for this purpose, as they are well-suited for modeling complex shapes and providing high accuracy in capturing stress and strain distributions.

A convergence analysis was conducted on the mandibular region using element sizes ranging from 5 mm to 0.5 mm. The resulting von Mises stresses exhibited significant variations with coarser meshes (5 mm to 3 mm), indicating solution instability in this range. As the mesh size decreased below 3 mm, the stress values stabilized, suggesting improved convergence. However, minor fluctuations persisted at finer resolutions (< 2 mm), likely due to numerical artifacts or localized stress concentrations. Given the model’s complexity — comprising multiple components with distinct material properties — a uniform refinement across the entire system was impractical. Thus, the study focused on the mandibular section, where a mesh size between 2 mm and 1 mm was selected as optimal, balancing computational efficiency with accuracy. Independent sensitivity analyses were performed for each component to determine appropriate element sizes.

The element size was a critical parameter in the discretization process and was tailored to each component’s size and complexity. The smallest component, the plastic cap, was modeled using an element size of 0.15 mm. This fine resolution ensured that intricate features and stress concentrations were accurately captured. Conversely, the largest component, the mandible, was assigned an element size of 1.5 mm. This coarser resolution was selected to balance computational efficiency with the need for an accurate representation of the overall geometry. Intermediate element sizes were used for other components, depending on their dimensions and the level of detail required. The discretization resulted in models with a total element count ranging from approximately 581,262 to 712,655, depending on the specific denture design. Correspondingly, the node counts varied between 896,711 and 1,098,823. These variations in element and node counts reflected differences in the geometric complexity and structural characteristics of each design. The use of a high number of elements and nodes ensured that the simulations were capable of delivering precise and reliable results, providing valuable insights into the performance and behavior of the denture designs under various loading conditions.

The material properties of the 3D model are summarized in Table 1. All materials are assumed to be homogeneous, isotropic, and exhibit linear elastic behavior.

**Table 1:** Mechanical properties of the materials used

Material	Mass Density	Young’s Modulus	Poisson’s ratio
Metallic parts of the denture	8.83 g/cm3	220 GPa	0.1–0.24
PMMA denture teeth, denture base	1.188 g/cm3	22.55 GPa	0.35–0.4
Cortical bone 1.2 mm outer layer of the jawbone	1.1 g/cm3	13.7GPa	0.3
Trabecular bone III inner part of the jawbone	0.5 g/cm3	1.6 GPa	0.2
Silicon Rubber	1.3 g/cm3	0.05 GPa	0.48

Although the simulated mandibular boundary conditions do not fully replicate physiological constraints, a simplified fixation approach was employed—applying a cut plane at the distal end to restrict all degrees of freedom, rather than modeling the temporomandibular joint. This simplification was uniformly implemented across all design variants to ensure consistent comparative analysis. Since the study focuses on *relative* performance evaluation among the four designs, the authors contend that this approach remains valid: any observed differences in mechanical response inherently arise from design variations rather than constraint artifacts. Thus, while the fixation method does not capture natural joint dynamics, it preserves the integrity of the design selection process by eliminating confounding variables in boundary condition effects.

**Fig. 2:**
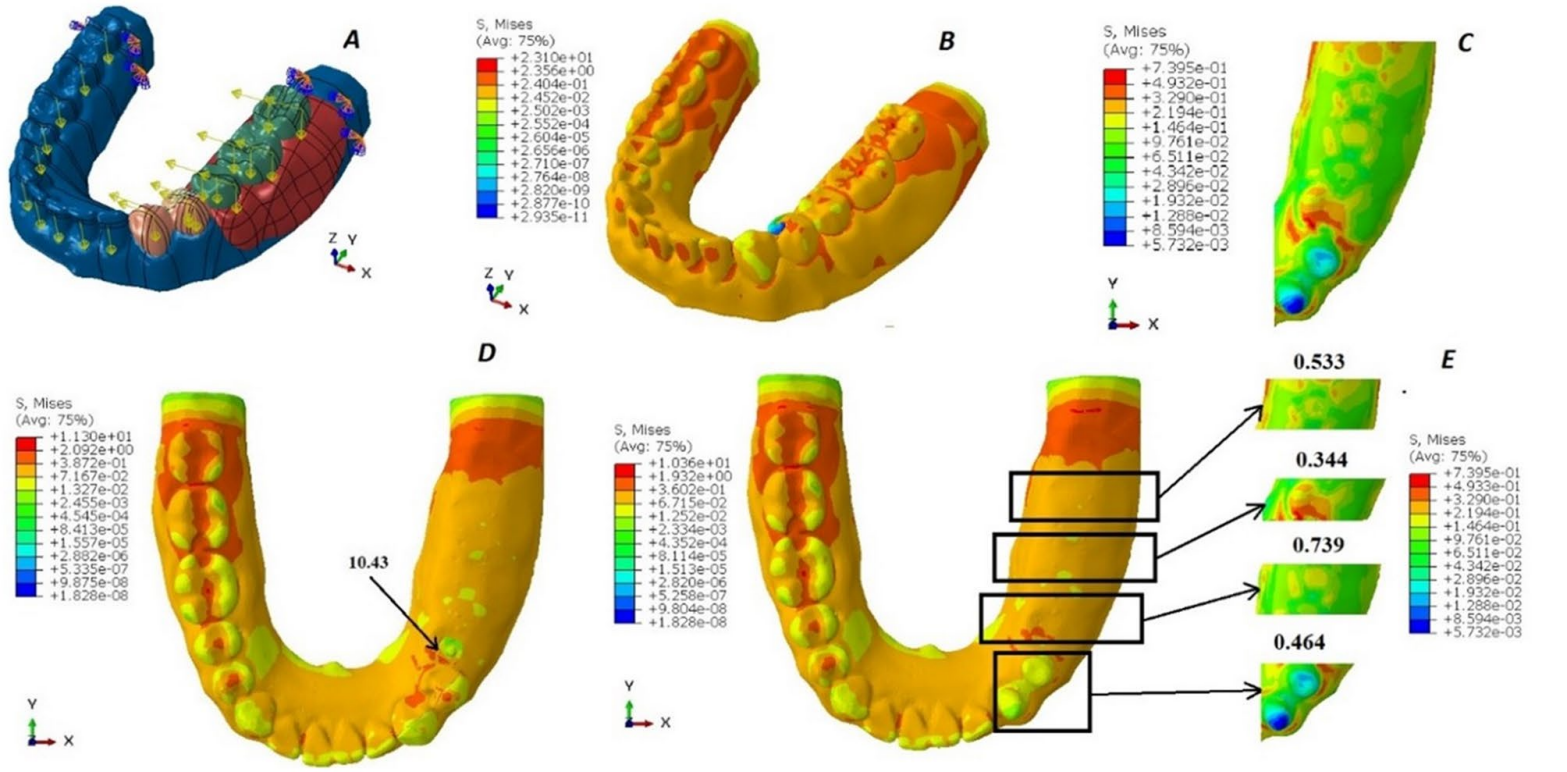
The von Mises stress levels of design 1 (OT cap). **A**: Force application to the 3D model. **B**: von Mises stress over the whole design. **C**: von Mises stress over the abutments and the residual ridge. **D**: von Mises stress under the attachment. **E**: von Mises stress over the abutments, the 2nd premolar, the 1st molar, and the 2nd molar area, respectively

**Fig. 3:**
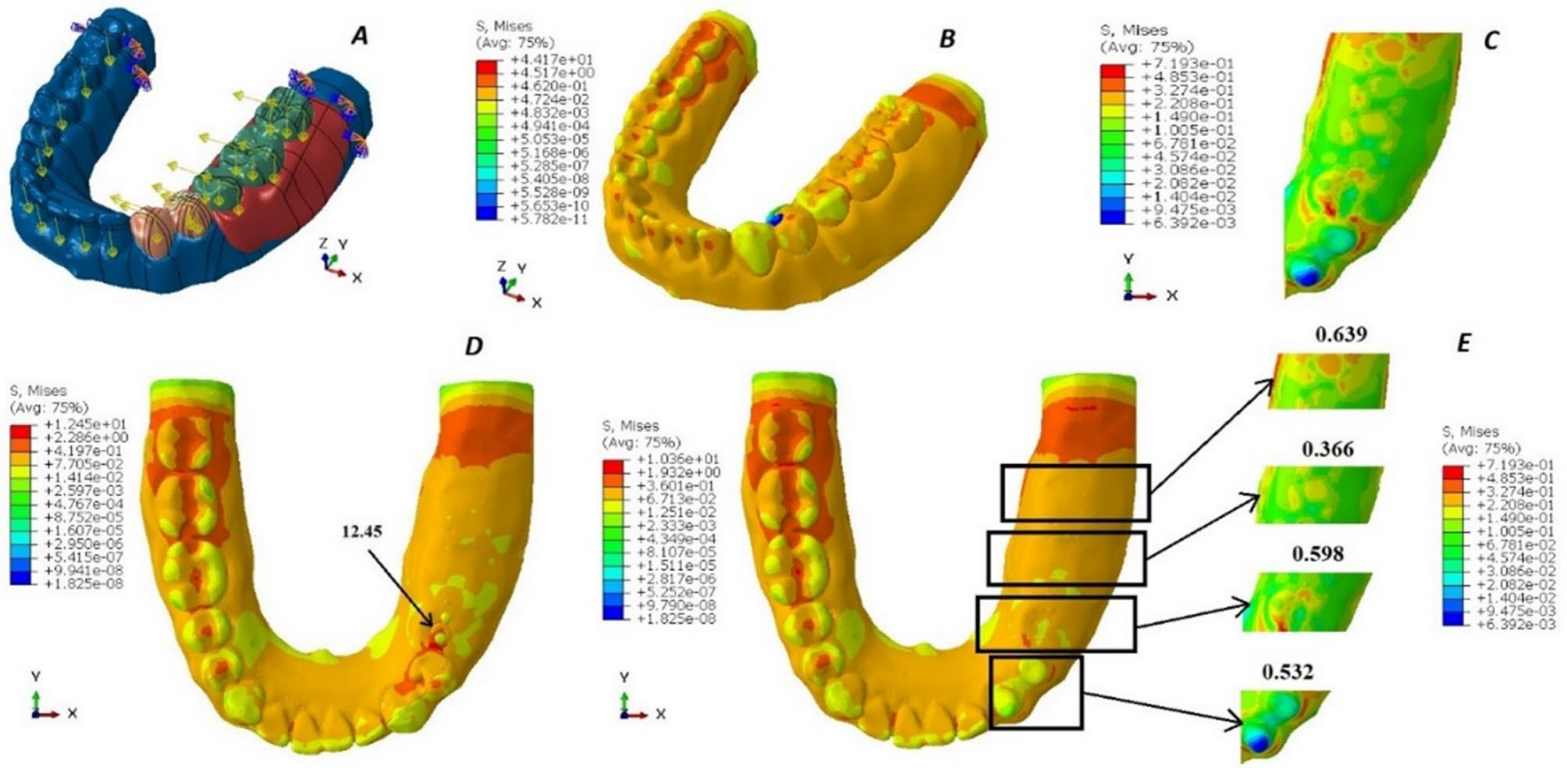
The von Mises stress levels of design 2 (double OT cap). **A**: Force application to the 3D model. **B**: von Mises stress over the whole design. **C**: von Mises stress over the abutments and the residual ridge. **D**: von Mises stress under the attachment. **E**: von Mises stress over the abutments, the 2nd premolar, the 1st molar, and the 2nd molar area, respectively

**Fig. 4:**
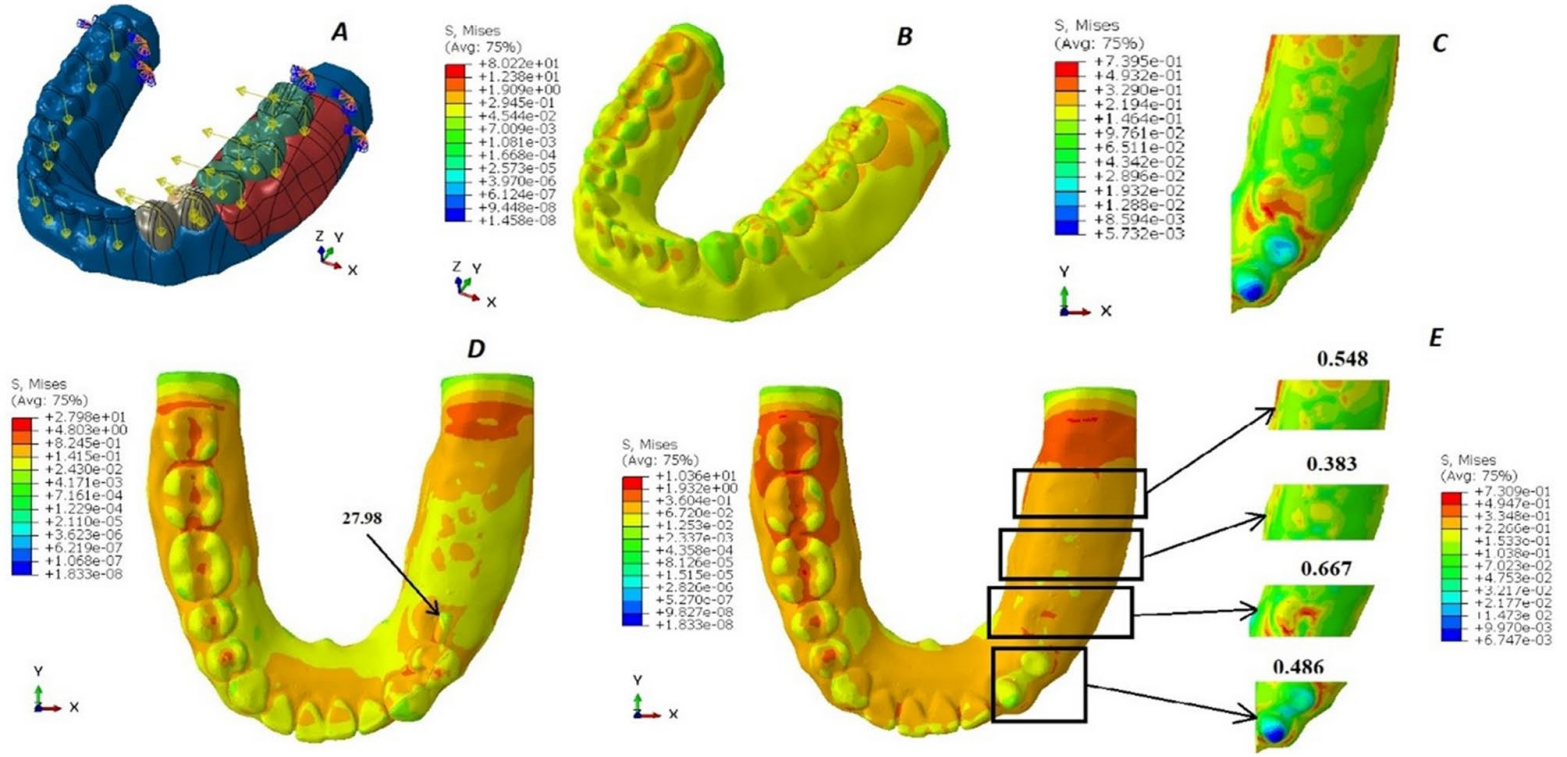
The von Mises stress levels of design 3 (mini bar). **A**: Force application to the 3D model. **B**: von Mises stress over the whole design. **C**: von Mises stress over the abutments and the residual ridge. **D**: von Mises stress under the attachment. **E**: von Mises stress over the abutments, the 2nd premolar, the 1st molar, and the 2nd molar area, respectively

**Fig. 5:**
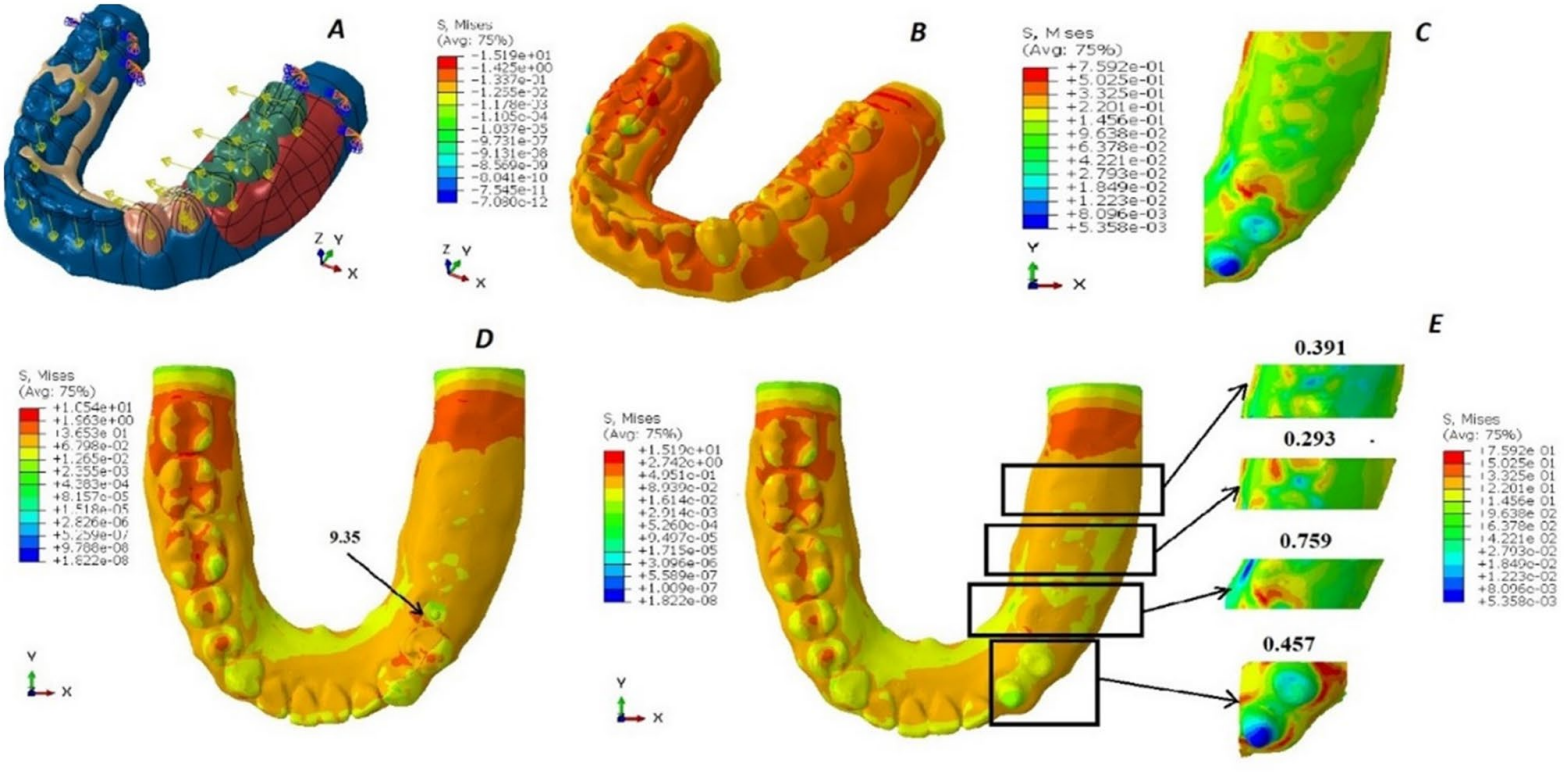
The von Mises stress levels of design 4 (conventional RPD). **A**: Force application to the 3D model **B**: von Mises stress over the whole design. **C**: von Mises stress over the abutments and the residual ridge. **D**: von Mises stress under the attachment. **E**: von Mises stress over the abutments, the 2nd premolar, the 1st molar, and the 2nd molar area respectively

Regarding the boundary conditions in the analysis, the mandible was fully constrained at both ends to prevent any movement or rotation in all directions, thereby simulating a fixed boundary scenario. On the edentulous side, specific load conditions were applied to replicate realistic chewing forces. The first molar and second molar on this side were subjected to vertical loads of 200 Newtons and tangential loads of 23.5 Newtons, mimicking the forces experienced during biting and grinding actions. Similarly, the first and second premolars were subjected to slightly lower vertical loads of 140 Newtons, along with tangential loads of 16.45 Newtons, reflecting the variation in force distribution along the dental arch. (Figures 2A, 3A and 4A, and 5A). These load conditions were chosen to accurately represent physiological masticatory forces under normal functional conditions [20].

## Results

**Fig. 6:**
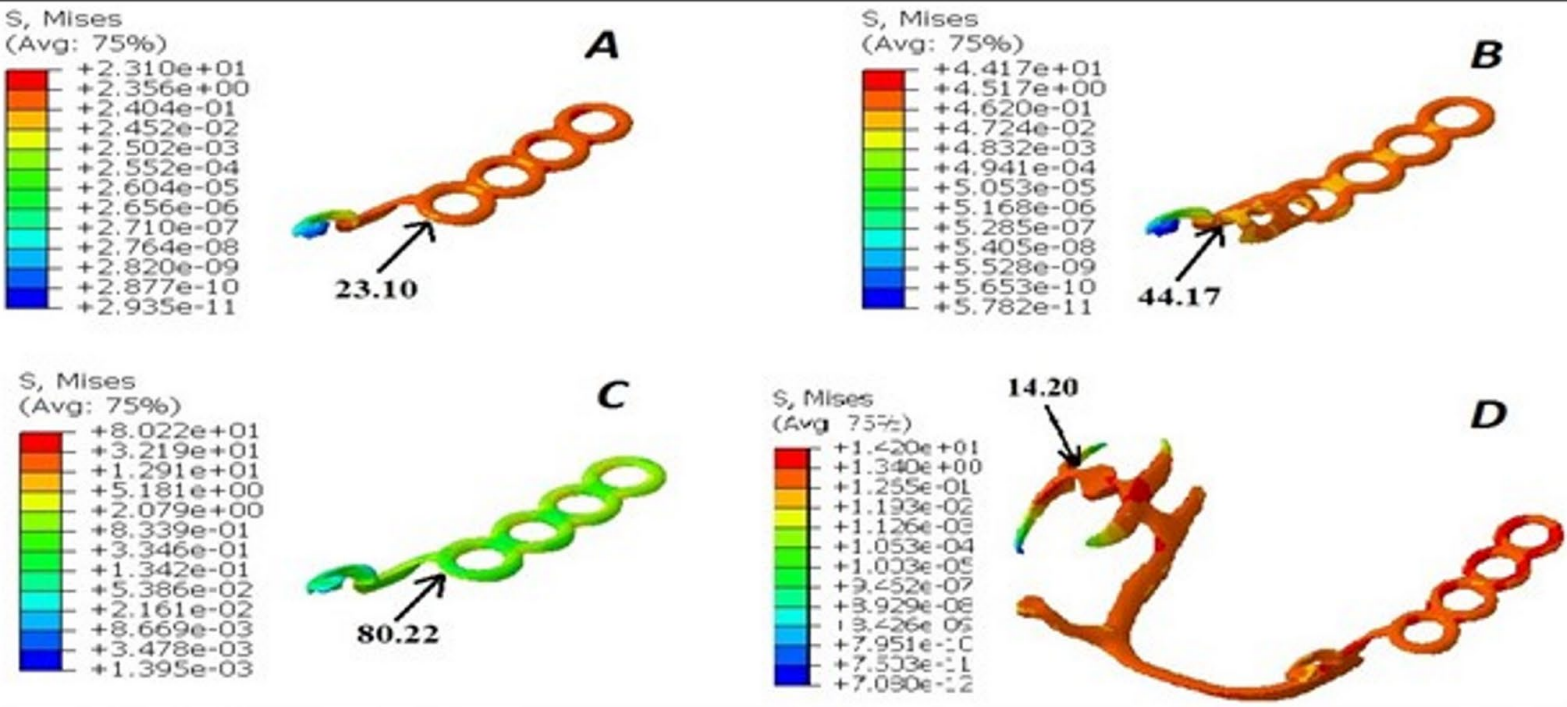
The maximum von Mises stress of each design. **A**: design 1 (OT cap), **B**: design 2 (double OT cap), **C**: design 3 (mini-bar), **D**: design4 (conventional RPD)

The von Mises stress levels induced around the abutments and in the region of the 2nd premolar, first molar, and 2nd molar are measured and compared across the four designs, as illustrated in Figs. 2, 3 and 4, and 5. Additionally, the maximum von Mises stress for each design is recorded, as shown in Fig. 6.

Comparison between von Mises stresses exerted by different attachment designs are presented in Table 2.

**Table 2:** Maximum von mises stresses (in MPa) in the four different designs (Considering both cortical bone and trabecular bone)

	Design 1 (OT cap)	Design 2 (Double OT cap)	Design 3 (MINI-BAR)	Design 4 (CONVENTIONAL)
Whole model	23.1	44.17	80.22	15.19
Mandible_Cortical	10.36	10.36	10.36	10.54
Mandible_Trabic.	0.18	0.18	0.18	0.19
Metal Crown	11.3	12.45	27.98	10.04
Porcelain Facing	2.61	3.66	4.22	2.33
Metal Framework	23.1	44.17	80.22	14.20
Acrylic Denture Based	15.32	21.27	10.22	15.19
Prepared Abutments	0.464	*0.532*	0.486	**0.457**
2nd premolar area	0.739	0.598	0.667	0.759
1st molar area	0.344	0.366	0.383	0.293
2nd molar area	0.533	0.639	0.548	0.391
Total load on edentulous area	*1.616*	1.603	1.598	**1.443**

BOld color indicates the lowest stresses, while italic color indicates the highest stresses

For the RPD attachment designs, the lowest von Mises stresses were observed in design 4 (conventional RPD), which recorded 15.19 MPa. This was followed by design 1 (OT cap) with 23.10 MPa, design 2 (double OT cap) with 44.17 MPa, and the highest von Mises stresses were recorded in design 3 (mini-bar) at 80.22 MPa.

The lowest Von-Mises stresses for metal crowns and the metal framework were observed in design 4 (conventional RPD), measuring 10.04 MPa and 14.20 MPa, respectively. This was followed by design 1 (OT cap) with values of 11.30 MPa and 23.10 MPa, respectively. Design 2 (double OT cap) showed higher stresses, recorded at 12.45 MPa and 44.17 MPa, respectively. The highest Von-Mises stresses were found in design 3 (mini-bar), with values of 27.98 MPa and 80.22 MPa, respectively.

However, in the acrylic denture base area, the lowest von Mises stresses were observed in design 3 (mini-bar) at 10.22 MPa, followed by design 4 (conventional RPD) at 15.19 MPa. Design 1 (OT cap) exhibited slightly higher stresses at 15.32 MPa, while design 2 (double OT cap) recorded the highest stresses at 21.27 MPa.

In the analysis of von Mises stresses on the prepared abutments, the lowest stresses were observed in design 4 (conventional RPD), followed by design 1 (OT cap) and design 3 (mini-bar). The highest Von-Mises stresses were recorded in design 2 (double OT cap).

Regarding the saddle, the lowest von Mises stresses were observed in design 4 (conventional RPD), followed by design 3 (mini-bar), then design 2 (double OT cap), with the highest Von-Mises stresses found in design 1 (OT cap).

## Discussion

Incorporating precision attachment in the RPD design has the advantages of superior esthetics through masking the metal clasps with minimal denture correction, resulting in more patients’ satisfaction compared to the use of clasped partial denture [21].

Epoxy resin was chosen to be the educational cast material as it has an elastic modulus value similar to that of jaw bones [22]. Digital fabrication of denture designs was selected to overcome the disadvantages of material discrepancies related to dimensional inaccuracy during the casting and processing of the dentures and ensure accuracy [23].

Splinting of the canine and the first premolar abutments was done by porcelain-fused-to-metal crowns for improved esthetics. Wide load distribution over more than one tooth resulted in enhanced support and consequently better prognosis of the dental prosthesis; moreover, buccolingual centralization of the attachment over the ridge aided in uniform stress distribution on the attachment-prosthesis system [24, 25].

Forces applied during finite element analysis were determined by previous studies to be about 400 Newtons (N) recorded in young healthy individuals at the 1st molar region during biting [26]. Additionally, the maximum unilateral biting force in the molars region is between 300 and 600 N [27]. At the anteriors region, the measured force is about 40%, and at the premolars region, it is about 70%. of the unilateral force recorded in the molar region [28]. However, in partial denture patients, the amount of biting force decreases by about half the normal range values [29, 30]. In this study, occlusal-like forces were applied to the 1st molar and 2nd molar of the edentulous side with 200 N vertical & 23.5 N tangential directions and applied to the abutments (canine & 1st premolar) with 140 N vertical and 16.45 N tangential directions.

In the present study, the stresses induced within the whole design and within the metal framework were found to be maximum in design 3 (mini-bar) and minimum in design 4 (conventional RPD). Comparing the stresses induced over the abutments with different attachment designs, it was observed that design 4 (conventional RPD) had induced minimum stresses over the abutment as it showed better denture stability with wide load distribution across the entire dental arch by the major connector crossing the dental arch. The cross-arch design aids in the resistance of horizontal and rotational forces resulting from mastication, leading to better stress distribution and protection of load-bearing structures from excessive stresses [31].

However, patients’ discomfort as a result of the large size of the denture arises the need to introduce different unilateral denture designs using extracoronal attachments and study their effect on the abutment and the supporting ridge regarding the induced stresses.

Regarding the unilateral designs: the minimum stresses on the abutments were found within design 1 (OT cap), followed by design 3 (mini bar), and the highest stresses were found in design 2 (double OT).

The OT cap is a resilient attachment designed to protect the abutment from torquing forces during mastication by directing most of the stresses to the abutment long axis; moreover, it permits some limited degree of rotational movement, so it dissipates the stresses applied to it toward the residual ridge with minimum effect to the abutment [32].

The lingual bracing arm incorporated in the studied attachments design counteracts adverse lateral forces, thereby reducing adhesion stress. It has also been reported that fabricating a ledge preparation on the lingual surface of the splinted crowns connected to the attachment and covering the abutments stabilizes and strengthens the denture and omits the need to cross the other side of the arch [33].

Stresses exerted by the mini bar attachment to the abutment teeth may be due to the vertical height of the mini bar attachment that leads to the creation of a vertical cantilever and, as a result, induces more stresses on the abutments [34].

Regarding double OT attachment design, there was a clinical study that claimed that the unilateral double OT attachment having two ends at two different levels (horizontal and longitudinal planes) allows for a better distribution of stresses applied under occlusal force and improved prosthetic stability [35–37]. However, the results of the present study showed that the highest stresses were found around the abutment, and this may be explained by a clinical trial done to compare different distal extension attachment retained RPDs and stated that attachment should be placed as close as possible to the abutment tooth in order to reduce tipping forces exerted on it. This is another reason that may explain why the double OT attachment exerted more force on the abutment compared to the OT cap in unilateral PD designs, as the double OT attachment has a longer arm of its male component attached to the splinted crowns, which contain two spheres located at different levels (unlike the OT cap, which has one sphere and a shorter arm), so when a load was applied to its end, which is located away from the abutment, torque force was created [38].

Comparing the stresses induced to the ridge, it was found that the minimum stresses induced to the ridge were found in design 4 (conventional RPD), and it was also explained by the cross-arch stabilization as mentioned before.

Regarding the unilateral designs: the minimum stresses on the ridge were found in design 3 (mini bar), followed by design 2 (double OT), and the highest stresses were found in design 1 (OT cap).

The orientation of the bar attachment is considered the main reason for the minimum stresses that are found in this design; the male part of the bar attachment was located parallel to the ridge and in a horizontal position to increase the attachment surface area to help reduce stresses transferred to the underlying supporting ridge [39]. In accordance with this, van Kampen et al., observed that ball attachments exhibited statistically more significant occlusal force values compared with bar attachment [40].

The double OT attachment showed lower stress transmission to the ridge compared to the OT cap attachment design, as it is considered to be a two-in-one design that contains two ball heads oriented in two different planes (vertical and horizontal planes). This unique position of the ball heads minimizes the stresses transmitted to the residual ridge. Double OT design allows it to be a shock-absorber-resilient attachment that distributes the masticatory load applied to it more favorably, enhances lateral stability, and improves distal support of the denture base. It allows for denture base movement; thus, it has stress-releasing properties [15].

The reason why the OT attachment showed the highest stress transmission to the ridge was explained and confirmed by another study done to compare different partial denture designs used for restoring Kennedy class II mandibular cases with distal implant and the results showed that the unilateral PD retained by OT cap group obtained the highest marginal bone loss around the distal implant, meaning that the unilateral OT cap attachment design cause more load dissipation toward the distal end of the ridge [24].

In contrast with these study results, a study was done using strain gauge analysis to compare the stresses transmitted to the supporting structures of Kennedy class II partial dentures retained by OT caps and mini-bars. It was found that modified OT attachment RPD exhibited the highest micro-strain on the abutments, while the mini bar attachment exhibited the highest micro-strain on the ridges and the lowest on the abutments [39].

In the current study it was noticed that the stresses found at the second molar area were greater than those found at the first molar area in all PD designs, and this was explained by a study done to evaluate the influence of saddle length on stresses transmitted to the residual ridge from unilateral RPD attached by double OT cap; it was noticed that, the stresses found at the second molar area were greater than that found at the first molar area in all PD designs and this was explained by a study done to evaluate the effect of saddle length on stresses transmitted to the residual ridge from unilateral RPD attached by double OT cap, it was found that, distal extension area showed more bone resorption than around the distal abutment in short and long saddle groups [41], which could be attributed to the splinting of the last two abutments that may eliminate stresses transmitted to their supporting bone [42]. In addition, the limited ability of the resilient retainers to equally disseminate the applied stresses to the artificial tooth professionally, leading to more direction of the load to the ridge [5].

For the *last decade*, the conventional bilateral RPD design was considered the most common treatment option *in Kennedy* class II cases for long time ago offering uniform stress distribution and cross arch stabilization over the entire arch, however, with introduction of attachment retained unilateral partial denture as more preferred treatment option over the bilateral design regarding patient satisfaction, smaller denture size with more tongue and tissue tolerance offered easier mastication and speech, it also offer superior retention, acceptable lateral stability, moreover, it solves many problems associated with the bilateral PD design such as increased soft tissue coverage that results in gingivitis, periodontitis and ends up with abutment *loss* [1, 43]. *Authors suggested that the trade-offs between designs*,* whether unilateral or bilateral PD*,* is done mainly according to the clinical situation. Conventional bilateral PD is still indicated when reduction of load on the abutments and the ridge is required*,* in cases of weak abutments and flat or knife-edge ridges; however*,* unilateral PD design retained by either OT cap*,* double OT cap*,* or minibar attachment is mainly indicated with well-developed ridges and relatively strong abutments*,* keeping in mind consideration as the different way of each attachment to dissipate the occlusal loads exerted on it*,* as mentioned in our study; OT cap*,* double OT cap and minibar attachment exerted 0.464 MPa*,* 0.532 MPa and 0.486 MPa on the abutments respectively*,* and exerted 1.616 MPa*,* 1.603 MPa and 1.598 MPa on the residual ridge respectively.*

Authors acknowledge that clinical trials are essential for evaluating patient variability and biological factors in translational research. However, in this study, finite element analysis (FEA) had provided a complementary approach -with time and effort saving- that allow for a simultaneous calculation of stress distributions under specific conditions that couldn’t be achieved clinically.

*This computational study employed finite element analysis to comparatively evaluate design variations under standardized loading and boundary conditions. While the model was not experimentally validated through strain measurements or cadaveric testing*,* this approach remains methodologically valid for assessing relative performance differences between designs. The authors recognize that absolute physiological accuracy would require experimental correlation*,* and we explicitly identify this as a key limitation in the revised manuscript (Section discussion– last paragraph). Future studies incorporating strain gauge validation or biomechanical testing would be valuable to confirm these computational predictions and enhance clinical translatability. The current findings should therefore be interpreted as demonstrating comparative rather than absolute biomechanical behavior.*

*The present study utilizes deterministic finite element analysis*,* which by definition employs fixed material properties*,* boundary conditions*,* and loading parameters to produce unique solutions for each design configuration. As this computational approach generates singular output values rather than probabilistic distributions or experimental replicates*,* statistical analysis was neither applicable nor required for interpreting the results. The absence of biological variability or measurement uncertainty in the simulation framework precluded the need for statistical validation methods commonly employed in experimental studies.*

## Conclusion

1. Attachment retained RPD with a conventional design having a major connector offered the least stresses applied for the abutment and the residual ridge.
2. OT cap attachment is considered the least attachment that exerts forces on the abutments when used in a unilateral design RPD, followed by the mini bar design.
3. *Double OT cap could be used with strong periodontal condition abutments*,* it suits patients with robust abutments as it concentrates more stresses on abutments compared by OT cap which concentrate more stresses on the ridge that might causes its resorption.*
4. It is not advisable to use OT cap attachment with flat or flabby or any weak forms of the remaining ridge, as it was detected that this type of attachment transmits most of the occlusal load to the ridge.

## Limitation of the study and recommendation for further investigations

- Masticatory-like forces applied to the different designs were in horizontal and tangential directions and of average magnitude; however, the amount and direction of the masticatory forces may differ intraorally.
- Moreover, the lack of statistical analysis between the designs in this study makes it possible to consider more future research to offer different unilateral removable denture designs as a possible treatment option.
- Boundary conditions, such as fixation of the mandible, in which a cut plane (instead of the joint) at the end of the mandible is used to prevent it from motion and rotation in all directions. Consistent with computational modeling standards, this study incorporated necessary simplifications—including isotropic material assumptions and static loading conditions—to enable systematic comparison of design variations. While these approximations do not fully replicate biological complexity, they provide a controlled framework for evaluating relative biomechanical performance. The authors acknowledge these limitations affect direct clinical extrapolation but maintain that the approach yields valid comparative data for implant design optimization. Future studies incorporating anisotropic properties, dynamic loading, and experimental validation would enhance physiological relevance while building upon these foundational computational insights.

## Data Availability

No data availability there are no data underlying the manuscript; all data were presented by figures and tables at the current study.

## Abbreviations

RPD: Removable Partial Denture
PD: Partial Denture
N: Newtons
FEA: Finite Element Analysis
